# A Consensus on Minimizing the Risk of Hyaluronic Acid Embolic Visual Loss and Suggestions for Immediate Bedside Management

**DOI:** 10.1093/asj/sjz312

**Published:** 2019-11-06

**Authors:** Greg J Goodman, Mark R Magnusson, Peter Callan, Stefania Roberts, Sarah Hart, Cara B McDonald, Michael Clague, Alice Rudd, Philip S Bekhor, Steven Liew, Michael Molton, Katy Wallace, Niamh Corduff, Sean Arendse, Shobhan Manoharan, Ava Shamban, Izolda Heydenrych, Ashish C Bhatia, Peter Peng, Tatjana Pavicic, Krishan Mohan Kapoor, David E Kosenko

**Affiliations:** 1 Monash University and Chief of Surgery at the Skin and Cancer Foundation, Victoria, Australia; 2 Griffiths University, Southport, Queensland, Australia; 3 St. Vincent’s Hospital, Melbourne, Australia; 4 Alfred Hospital, Prahran, Victoria, Australia; 5 University of Melbourne, Department of Paediatrics and Director of the Laser Unit, Department of Dermatology, Royal Childrens’ Hospital, Parkville, Victoria, Australia; 6 Alfred Hospital, Melbourne, Victoria, Australia; 7 Northwestern University, Chicago, IL; 8 Department of Plastic Surgery, Fortis Hospital, Mohali, India

## Abstract

**Background:**

Hyaluronic acid fillers have a satisfactory safety profile. However, adverse reactions do occur, and rarely intravascular injection may lead to blindness. Currently there is no internationally recognized consensus on the prevention or management of blindness from hyaluronic acid filler.

**Objectives:**

The authors sought to give guidance on how to minimize the risk and optimize the management of this rare but catastrophic adverse reaction.

**Methods:**

A multinational group of experts in cosmetic injectables from multiple disciplines convened to review current best practice and develop updated consensus recommendations for prevention and bedside intervention if visual loss occurs after cosmetic injection of hyaluronic acid filler.

**Results:**

The consensus group provided specific recommendations focusing on the consenting process, prevention, and early management of visual impairment related to intravascular hyaluronic acid filler injection.

**Conclusions:**

Although visual loss due to filler injections is rare, it is important that both patient and physician be aware of this risk. In this paper the authors describe methods and techniques available to reduce the risk and also document suggested initial management should a clinician find themselves in this situation.

**Level of Evidence: 5:**

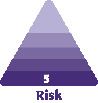

## 抽象

**背景:**

透明質酸填充劑具有令人滿意的安全性。但是，確實會發生不良反應，很少進行血管內註射可能導致失明。當前，關於透明質酸填充劑的失明的預防或管理尚無國際公認的共識。

**目標:**

作者試圖就如何減少這種罕見但災難性的不良反應的風險和優化管理提供指導。

**方法:**

來自多個學科的化妝品注射劑國際專家小組召集起來，審查當前的最佳實踐，並為透明質酸填充劑化妝品注射後出現視力下降提出預防和床旁干預的最新共識建議。

**結果:**

共識小組提供了針對與血管內透明質酸填充劑注射相關的視覺障礙的同意過程，預防和早期治療的具體建議。

**結論:**

儘管很少發生因填充劑注射引起的視力喪失，但重要的是患者和醫師都應注意這種風險。在本文中，作者描述了可用於降低風險的方法和技術，還提出了建議，如果臨床醫生髮現自己處於這種情況，則應進行初步治療。

**Figure 1.**
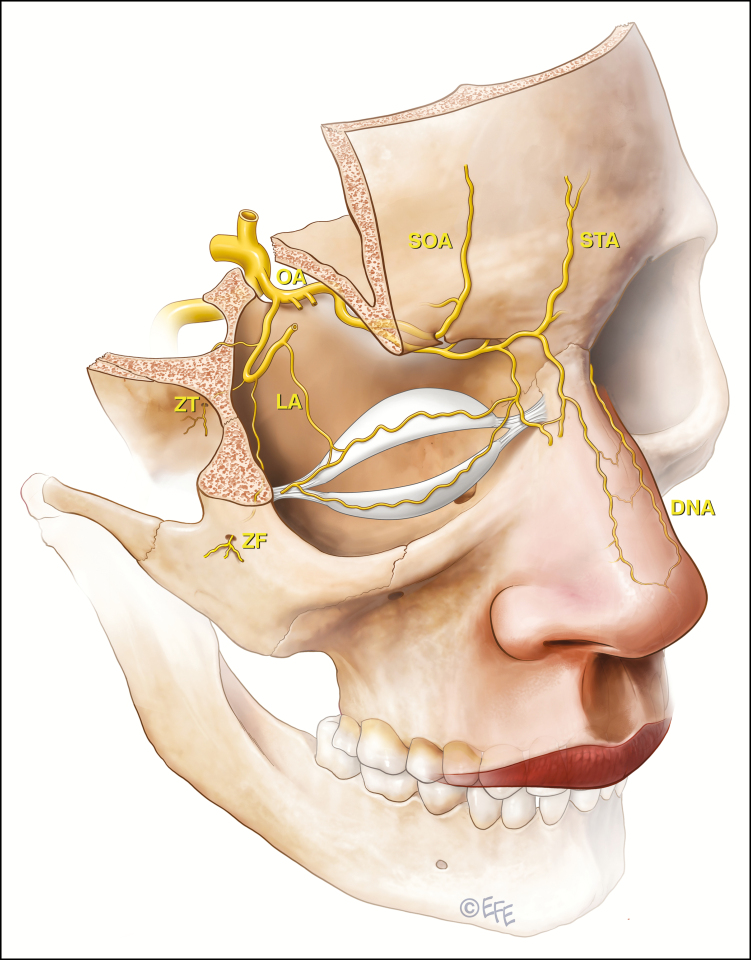
The ophthalmic artery system arises from the internal carotid artery and the danger zones are associated with the end vessels that supply facial tissues or via anastomotic connections of the external carotid system with the ophthalmic end vasculature. The ophthalmic artery (OA) end vessels are the supraorbital (SOA), supratrochlear (STA), dorsal nasal artery (DNA), and lesser recognized zygomaticofacial (ZF) and zygomaticotemporal (ZT) that both arise from the lacrimal artery (LA). Not demonstrated on this illustration and with unknown relevance to filler induced blindness is the anterior ethmoidal artery, which has a terminal cutaneous branch, the anterior nasal artery. This vessel enters the nasal dorsum between the nasal bone and upper lateral cartilage.

**Figure 2.**
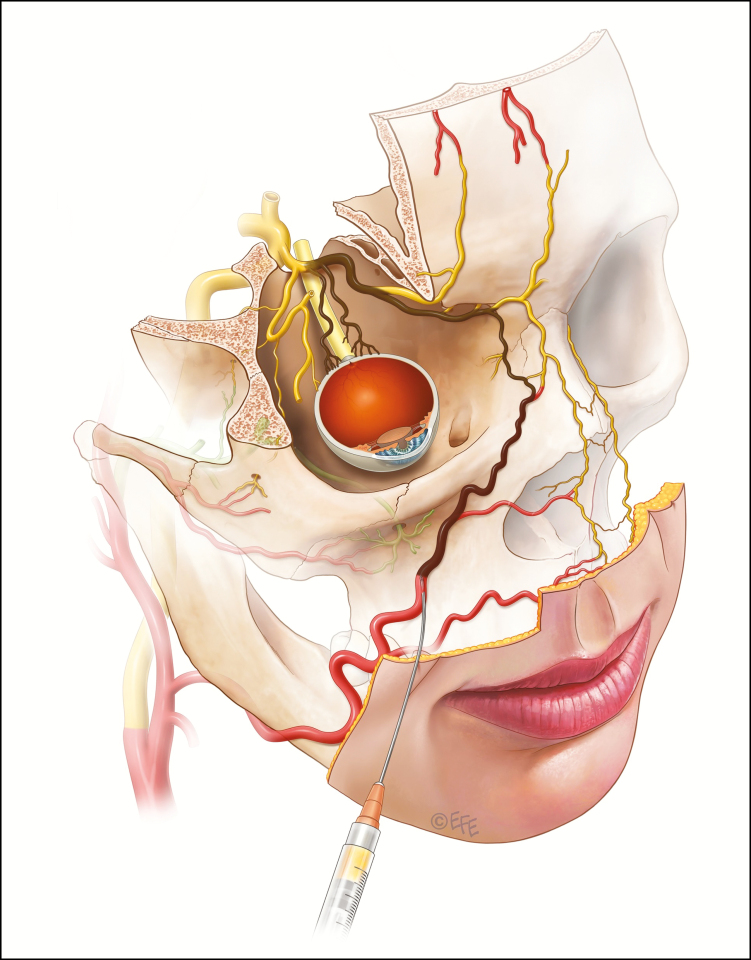
It is presumed that injectable filling agents may cause blindness if a column of filler is pushed against the prevailing arterial blood pressure of the ophthalmic artery and gains access to the central retinal arterial system and/or the ciliary vessels. An embolus is carried forward by the usual direction of flow on release of the plunger. The locations of greatest risk appear to be the forehead, glabella, and nasal dorsum. The nasolabial fold region is the area of next greatest risk.

Blindness following the utilization of tissue fillers is a rare but devastating complication in the use of injectable filling agents.^1-3^ It has occurred with a variety of agents including hyaluronic acid, fat, and calcium hydroxyapatite with the employment of both cannulae and needles.^4-6^ It is presumed that these products enter the vascular circulation of the eye via intravascular injection. This may involve either the ophthalmic artery end vessels, supplying the facial tissues (well-known supraorbital, supratrochlear and dorsal nasal as well as the lesser known branches of the ophthalmic artery via the lacrimal artery - the zygomaticofacial and zygomaticotemporal arteries that emerge through facial foramina towards the skin). This intravascular injection may also involve anastomotic connections of the external carotid artery with the ophthalmic end vasculature. Most notabe examples are via the angular branch of the facial artery but also the transverse facial via zygomaticofacial, superficial temporal frontal branch and deep temporal via the zygomaticotemporal arteries (Figure 1). A column of filler, pushed via the filler syringe plunger against the prevailing arterial blood pressure of the ophthalmic artery, would appear to be a necessary prerequisite to visual loss.^7,8^ Once the column of filler has filled the ophthalmic artery (or its anastomoses) past the origin of the central retinal artery and ciliary vessels, these vessels are essentially occluded (Figure 2). A release of the plunger pressure may also allow the filler to obstruct these vessels carried forward by the usual blood pressure and direction of flow. It has been suggested that at least 0.04 to 0.12 cc needs to be injected to backfill the supratrochlear arteries to the origin of the central retinal artery.^9^ Retrograde flow from the supratrochlear artery into the ophthalmic artery was successfully shown in a cadaveric study. In this paper, 6 fresh cadavers had their arterial systems pressurized somewhat less than 120 mmHg with a plasma-based perfusate. In 3 of 6 cadavers, retrograde flow into and filling the ophthalmic artery was shown following cannulation of the superficial branch of the supratrochlear artery (average depth, 1.5 mm; average diameter, 1.42 mm) and injection of a hyaluronic acid filler. This retrograde filling required an average injection pressure of 166.7 mmHg (range, 160-180 mmHg).^10^

The degree of subsequent visual disturbance would relate to the nature of the filler: the volume injected, its cohesivity, particle size, and the vessels affected. Total obstruction of the ophthalmic artery would tend towards visual loss, ptosis, and ophthalmoplegia, whereas isolated central retinal artery occlusion would tend towards visual loss alone. Branch retinal artery occlusion may cause a partial visual loss.^11,12^ Combinations of these patterns can occur.^13,14^ Some visual loss patterns would be more amenable to reversal than others, and hyaluronic acid would be the only injectable agent potentially reversible at all via intervention.^12,15-17^ The adverse event of stroke that may occur in about 25% of those with visual disturbances following fillers was also discussed by the group but was included in only one of the consensus statements on examination.^1^

## Key Points

1. A column of filler is probably required to induce embolic visual loss from hyaluronic acid injection
2. The degree of visual loss may relate to the volume injected, its cohesivity, particle size, and vessels affected
3. The exact embolized vessels within the orbital system will dictate the clinical pattern
4. Cerebral infarction may occur in 25% of patients with embolic visual loss

## METHODS

A consensus meeting was held on the September 23, 2018 in Melbourne, Australia and comprised a consensus group of 22 members from Australia, New Zealand, South Africa, India, USA, and Taiwan.

A number of prepared questions, designed by the lead author (G.J.G.), were introduced to the consensus meeting. Options were then put to the group for a vote, allowing the development of a consensus statement when greater than 75% agreement was reached. Anything less than 75% agreement was not deemed to be a consensus.

After the consensus meeting, further minor refinements were allowed in a discussion forum. This discussion did not alter the accepted consensus statements but offered clarification and refinement. These changes were then accepted unanimously by the group.

After the consensus statements were finalized, 5 ophthalmologists and 2 interventional radiologists were consulted regarding their opinion on the outcomes of the meeting and the consensus statements. These opinions were taken back to the group for discussion and further refinements were made, although none of the consensus statements were substantively altered and no agreements affected.

The consensus questions were (Table 1):

1. Should the consent document given to a prospective patient include a discussion about the possibility of visual loss?
2. What preventive strategies should be undertaken by the practitioner to decrease the incidence of post-filler visual loss?
3. Are there areas of increased risk that require more training and more supervision?
4. What should be documented at the bedside if visual loss is suspected or apparent?
5. If hyaluronic acid was the substance being used, what role does hyaluronidase have in the management of the visual loss?
6. Should the clinic anticoagulate the patient at the bedside with aspirin or other agents?
7. Should the clinic attempt maneuvers to decrease intraocular pressure at the bedside or during transport?

**Table 1.** Consenting for the Possibility of Filler-Induced Complications

This consenting document is based on the following principles
The patient has a right to know about the possibility of visual loss
Even though it is rare (estimated ≤1:100,000), it is potentially life-changing
The patient may not have proceeded with treatment had they known of this possibility
The consenting practitioner should consent the patient to allow remedial action to be taken if an intravascular event is suspected

## RESULTS

The following were the results of these discussions.

### Question 1: Should the Consent Document Given to a Prospective Patient Include a Discussion About the Possibility of Visual Loss?

The consensus group considered that the consent document should ideally state:

- There is a small but definite risk that injection of fillers may injure a facial blood vessels
- Most times this just produces bruising and is not overly significant
- However, rarely the filler will enter a blood vessel and produce an event that may result in skin and tissue loss if not treated urgently
- Very rarely, estimated at 0.001% (less than 1 in 100,000 injection syringes used), blindness has occurred from filler being injected in a vessel, which may be irreversible. Also, very rarely there may be a risk of stroke from an injection into a vessel.
- If the practitioner notices problems (of blindness or pending tissue necrosis) at the time of the procedure or subsequent to the procedure it is essential that they are allowed to dissolve this material or take other remedial measures, and the patient should consent to this approach prior to the procedure

The group acknowledged that the estimated incidence of this complication is an approximation. The real incidence of blindness is probably underreported but has recently been suggested to be 146^18^ to 190^12^ cases globally. This number includes fat transfer cases, which make up a significant proportion of reported cases, although most recent cases appear to be hyaluronic acid induced.^17,19^ Most cases have involved visual loss in 1 eye. Visual loss in both eyes has also been reported.^20^ The group utilized 300 cases as its numerator. The denominator was set at 30 million syringes although the real figure is probably significantly larger than this because over 20 million syringes of hyaluronic alone (not including other autologous and nonautologous fillers) are now being utilized per year. Thus, the figure is deliberately set at the upper limit of likelihood.

It was deemed important that the patient consents to intervention and management of the vascular occlusion to limit the complication if possible. This was agreed to unanimously (Table 1).

### Question 2: What Preventive Strategies Should Be Undertaken by the Practitioner to Decrease the Incidence of Post-Filler Visual Loss?

Are there strategies that may be employed on a regular basis that may lessen risk (Table 2)? There were 9 consensus points decided on by the group. This required the most discussion both within the group and in refinement group discussion following the meeting.

1. Understand the safest depth of injection in any given area^21-24^
2. Inject VERY slowly and with low extrusion pressure^21,25^
3. Cannulae are considered by many to be a safer alternative to needles in certain areas including the brow and lateral and anterior cheek. They are not considered safer for nasal injection. Smaller gauge cannulae (less than 25 gauge) may behave somewhat like needles in terms of their ability to pierce blood vessels.^26,27^
4. Consider utilizing local anesthetic with epinephrine at cannula entry points and within the injection field to constrict local vessels. When utilizing local anesthetic with epinephrine it may be worthwhile observing the patient after injection to ensure the vasoconstrictive effect resolves to avoid confusion with intravascular injection of filler.
5. Consider directing the needle/cannula perpendicular to primary axial vessels in the anatomical region to reduce the likelihood of vessel cannulation.
6. Micro-boluses should be injected in small aliquots (<0.1 mL).^7^
7. Move the needle in the chosen plane at all times when delivering micro-boluses, even if only in small amplitude movements.^21,24^
8. Consider ensuring the direction of injection is away from the eye in higher risk areas such as nose, glabella, and nasolabial fold.
9. There is currently no evidence to support aspiration as a safety measure.

**Table 2.** Regions of the Face and Their Relative Risk of Blindness and Visual Complications

Risk	Regions
Low	Jawline and marionette, lateral cheek (lateral to a vertical line through lateral canthus), sub-malar, preauricular, chin augmentation
Moderate	Lips, perioral region, anterior cheek (between a vertical line through lateral canthus and mid-pupillary line)
High	Temples, nasolabial folds, tear troughs, peri-orbital, medial cheek (between mid-pupillary line and side of nose)
Very high	Glabella, nose, forehead

Among these points, aspiration particularly warranted much discussion. The group felt that there is currently no evidence to support aspiration as a safety measure. Review of the literature and discussion among this group of experts illustrate that this is an unreliable and impractical technique. Numerous studies have shown a high false-negative rate with a positive aspiration in in vitro studies ranging from only 33% to at best 53% of attempts.^27-30^ Aspiration results are profoundly influenced by needle diameter and length, the rheology of the filler being injected, whether the needle is empty or already contains filler in its lumen, syringe dimension, blood pressure, and, most importantly, degree of negative pressure and the time this pressure is maintained for. Evidence that filler material tracks distant from the needle tip to a more superficial plane further renders a negative aspiration result unhelpful.^31,32^

A negative aspiration (needle in vessel but no aspiration achieved) may also risk giving the injector a false sense of security to proceed with a risky injection, and a positive aspiration (blood on aspiration) will only be relevant for the instant the needle is in that position. After a false negative aspiration, an injector may be encouraged to proceed to inject through a needle tip that is being held steady but may be within a vessel. This could permit and encourage bolus injection of a cohesive column of embolic material to be delivered into the vascular system. Thus, we cannot recommend aspiration and in fact recommend against it.

This group believes that a safer technique is to inject product very slowly through a constantly moving needle even given that they may pass momentarily through vessels. If the injector combines slow injection and low extrusion pressure with this continuing movement then any intravascular product delivery should be minimal. This will in turn minimize the amount of material delivered into any potentially encountered vessel to less than the threshold required for any meaningful and problematic embolism.

It is likely that all filler injections pass through many blood vessels during product delivery in all areas of facial injection (as evidenced by bruising). In certain areas such as lips, practitioners will rarely resort to aspiration despite this region’s vascularity. In these areas most professionals, whether aware of it or not, will utilize these principles of slow injection, low extrusion pressure, and movement together with an anatomical understanding instinctively without resorting to aspiration. The same principles should apply in all sites of facial injections.

To apply this safer technique of slow injecting through a moving needle to create a “bolus” (where the aspiration test is most often employed), minimal oscillating movements of the injecting hand will place multiple microboluses in the same area, cumulating in a bolus deposit of product in the desired plane while adhering to maximal safety in practice. We also suggest angling the needle (preferably with bevel down) because this may facilitate the ability to employ these minimal oscillating movements within the same plane.

Many of these recommendations are similar to those presented in a recent blindness review.^18^ Additionally, the authors of this review suggested being especially cautious in those patients who previously underwent surgery and in application of digital pressure to occlude major periorbital vessels as additional exercises in safety. Notably, most of the cases of blindness between the authors’ 2 reviews in 2015 and 2018 involved the nasal region (56.3%), glabella (27.1%), and forehead (18.8%), equating to our grade 4 regions of highest risk (consensus point 3) and further substantiating our consensus point 8 above. This was agreed to unanimously.

### Question 3: Are There Areas of Increased Risk That Require More Training and More Supervision?

Should we, as a consensus group, suggest grading regions of concern by risk and advise the required level of access to medical care (Table 2)?

- This is predicated on 2 facets of practice: the amount of teaching and experience of the practitioner and the ready access to medical care if required.
- Should teaching and learning deal with regions of least risk first, moving on to more difficult regions as experience and knowledge increases?
- Are there regions that should not be treated without medical clinic status and/or access to medical assistance?

We graded regions into 4 groups dependent on risk associated with these principles (Table 2).

#### Grade 1

Grade 1 involves a low likelihood of serious intravascular injection that may lead to visual embolic events. These were thought to be excellent regions for a beginner because they define lower facial shape, which is important in sexual dimorphism (defining masculinity and femininity) and attractiveness. It would possibly be a better area for the beginner than the more traditional but less safe “starting points” of lips and nasolabial folds.

- Regions of lower risk
- Should only be treated by those with adequate training
- Utilizes up-to-date knowledge of regional anatomy and safest practice
- Performed in a clinical setting
- Jawline and marionette, lateral cheek (lateral to a vertical line through the lateral canthus), sub-malar, preauricular, chin augmentation

#### Grade 2

Grade 2 or moderate likelihood of serious intravascular injection that may lead to visual embolic events was considered to be the next safest set of regions that should be taught and attempted by new injectors. This was felt to be similar in requirements but possibly after the practitioner had initial training and experience in filler utilization and injection techniques.

- Regions of moderate risk
- Should only be treated by those with adequate training
- Utilizes up-to-date knowledge of regional anatomy and safest practice
- Performed in a clinical setting
- Lips, perioral region, anterior cheek (between a vertical line through the lateral canthus and the mid-pupillary line)

Lips and the perioral area are considered high incidence areas for intravascular injection and embolism but only moderate likelihood of visual loss.

#### Grade 3

Grade 3 or high-risk areas are those with significant likelihood of serious intravascular injection that may lead to visual embolic events. These higher risk regions require strict adherence to best technique with good anatomical knowledge of depth of injection and product placement. They also require the practitioner to strictly adhere to the tenets outlined in consensus point 2 regarding avoidance maneuvers for intravascular injection.

- Regions of high risk
- Should only be treated by those with adequate training
- Utilizes up-to-date knowledge of regional anatomy and safest practice
- Performed in a clinical setting with rapid access available to medical personnel if required
- Temples, nasolabial folds, tear troughs, peri-orbital, medial cheek (between the mid-pupillary line and the side of the nose)

#### Grade 4

**Figure 3.**
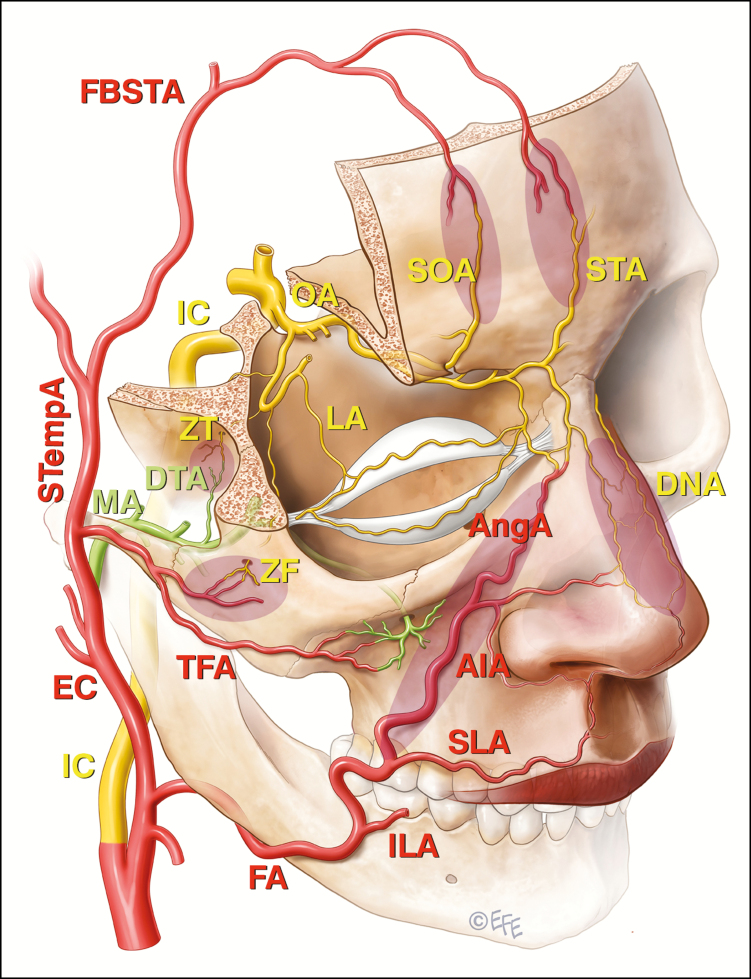
The communications between the external carotid system and the end branches of the ophthalmic artery (OA) that are implicated in filler induced blindness are shaded. These include the angular branch (AngA) of the facial artery (FA), the transverse facial artery (TFA) arising from the superficial temporal artery (STempA), and the deep temporal branch (DTA) of the maxillary artery (MA) in shaded zones as indicated. The frontal branches of the superficial temporal artery (FBSTA) pass anteriorly into the forehead and will eventually anastomose with the supraorbital (SOA) and supratrochlear arteries (STA), but the FBSTA have not been directly implicated in blindness following filler injections. Other vessels indicated are internal carotid artery (IC), external carotid artery (EC), inferior labial artery (ILA), superior labial artery (SLA), inferior alar artery (AIA**),** dorsal nasal artery (DNA), zygomaticofacial artery (ZF), zygomaticotemporal artery (ZT), and lacrimal artery (LA).

Grade 4 regions were considered to be those at highest risk of inadvertent intravascular injection and possible risk of blindness.^18,19,24^ They represent regions with direct injection access to branches of the ophthalmic circulation or, in the case of the nose, a region with widespread anastomoses between branches of the external carotid and ophthalmic division of the internal carotid arteries (Figure 3). It was felt that before injecting Grade 4 regions, the practitioner requires extensive experience and training with understanding of the relevant anatomy and possible variations in that anatomy. It also requires strict adherence to the tenets of consensus point 2 regarding avoidance of intravascular injection.

- Regions of greatest risk of serious intravascular injection that may lead to visual embolic events
- Should only be treated by those with extensive experience
- Requires comprehensive training
- Utilizes up-to-date knowledge of regional anatomy and safest practice
- Performed in a medical clinic
- Glabella, nose, forehead

This was agreed to unanimously (Table 2).

### Question 4: What Should Be Documented at the Bedside if Visual Loss Is Suspected or Apparent?

If the practitioner suspects visual loss, what should be done at the bedside? It was felt that this is a true vascular emergency, and, as with a cardiac or neurological vascular occlusion, it should be treated as such. The priority should be urgent referral to expert care with additional actions suggested below. This recommendation took into account postmeeting discussion with the ophthalmologists and interventional radiologists.

The recommendations included:

- Seek help from in-clinic colleagues and aim to transfer the patient at the earliest opportunity to external personnel such as an emergency physician from a specialist eye hospital with experience in treating this or similar conditions, or otherwise an experienced ophthalmologist
- Document the time of the beginning of the vascular event
- Document visual deficit if able to assess. This step should not cause delay in transfer
- If assessing visual loss, ask the patient to read text, assess number of fingers held up in front of the affected eye, and, if unable to do that, assess detection of hand movement
- One eye should be assessed at a time
- Assess pupillary reactions, eye movements
- Questioning should assess history of migraines if not ascertained beforehand
- Providing it does not delay transfer, basic neurological assessment should be completed, including speech, hand, arm, and leg movements and level of consciousness (NB 25% rate of associated stroke has been reported)^1^
- Assess any associated cutaneous signs of impending skin necrosis
- Take photographs if time permits or a short video, possibly via a phone or tablet, if this is the most expeditious method

This was agreed to unanimously (Table 3).

**Table 3.** Suggested Assessment of Patient After Visual Loss Is Suspected

Bedside management	
Transfer the patient at the earliest opportunity for definitive diagnosis and treatment	While awaiting transfer, ascertain 1. Visual impairment, eye movement, and pupillary changes 2. Signs of cerebral infarction 3. Signs of vascular cutaneous compromise 4. Take photographs

### Question 5: If Hyaluronic Acid Was the Substance Being Used, What Role Does Hyaluronidase Have in the Management of the Visual Loss?

What should every clinic know about hyaluronidase and what should they be expected to do with this agent (Table 4)?

**Table 4.** Suggestions for Hyaluronidase Availability and Use in Advent of Suspected Visual and Associated Cutaneous Impairment

Hyaluronidase	Expectation
Clinic handling	Every clinic should have hyaluronidase on hand and be able to inject this agent
Local use	If visual loss is diagnosed, clinician should consider injecting high-dose hyaluronidase at area where injection appeared to induce visual loss and to area of any suspected cutaneous vascular compromise
Regional use	If clinician is comfortable with technique, inject high-dose hyaluronidase at supraorbital, supratrochlear, or other ophthalmic artery branches
Retrobulbar and peribulbar use	Only if clinician is certain of diagnosis and has requisite knowledge and skill should retrobulbar or peribulbar injection be attempted

Hyaluronidase was only considered useful if hyaluronic acid filler was known to be the cause of the visual loss. It again should be stressed that the person available with the highest level of expertise should be handling this stage of treatment, and any treatment should not delay appropriate transfer. Treatment should be extremely timely because time to reverse the blindness is short. There is very limited evidence to support intervention but both supratrochlear,^17^ supraorbital,^16^ and dorsal nasal vascular access^33^ treatments and retrobulbar injections^15^ have had some limited success.

- Every clinic must have hyaluronidase available for immediate utilization in case of any intravascular event
- Every clinician utilizing hyaluronic acid filler must have reconstituted or be able to reconstitute hyaluronidase
- In Australia and New Zealand, the hyaluronidase Hyalase (Sanofi-Aventis Australia Pty Ltd) is available as 1500 units as a powder for reconstitution. We recommend that a minimum of 7500 units in total should be available on site and maybe even more than this may be required in certain circumstances. Other jurisdictions will have other forms of hyaluronidase with different units per vial, but units are interchangeable between agents.
- In the case of visual impairment, high-dose (1500 units in 2 mL xylocaine 1%) hyaluronidase should be injected where the hyaluranon filler was placed and any other areas demonstrating signs of impending skin necrosis.
- Injection of high-dose hyaluronidase (1500 units in 2 mL xylocaine 1%) should be performed at the supraorbital margin specifically in the location of the supratrochlear artery (14 mm from midline beneath the medial brow creases) and/or supraorbital notch or foramen (25 mm from midline) or targeting other branches of the ophthalmic arterial system if the practitioner feels comfortable with this technique.

Peribulbar or retrobulbar injections should only be performed if the operator has expertise in this injection technique and feels confident in the diagnosis. Various injection volumes and number of units have been attempted from 150 units^15^ to over 1000 units.^34^ In other geographical regions, different or multiple forms of hyaluronidase will be commercially available. The dosing of other forms of hyaluronidases will not correlate directly with this protocol.

Concern is sometimes raised about the utilization of hyaluronidase in high doses and its possible effects on tissue. We are unaware of studies suggesting that high concentrations produce any permanent tissue damage in vivo or in vitro. This issue was addressed as a discussion, observing effects in a monolayer of primary human keratinocytes and primary human fibroblasts with no adverse effects on wound healing, although these observations were not published.^35^ This showed no adverse effects on these cells. The influence of hyaluronidase on wound healing was also investigated in a study utilizing a suction blister method in a prospective, placebo-controlled, double-blind, intra-individual comparison study of 20 participants. No retardation of wound healing or other relevant adverse effects were observed.^36^ Hyaluronidase has been found to be safe in its established utilization in the management of extravasation of cytostatic drug infusion,^37^ for faster rehydration in pediatric patients,^38^ and as an excipient in the subcutaneous injections of anti-HER2, anti-CD-20 monoclonal antibodies.^39^ Practitioners should feel comfortable to inject high-dose hyaluronidase if required. Furthermore, it would seem that once hyaluronidase is injected into the patient, it acts as a trigger to set off fibroblast hyaluronic acid production.^40^

If one lowers the concentration of hyaluronidase, this would necessitate increasing the injected volume to attain a sufficient dose to dissolve hyaluronic acid fillers. In the context of the retrobulbar or peribulbar injection, high volume may add to the risk of tamponade. Tamponade from allergy and bleeding have caused issues with vision.^41-43^ Hence, the ophthalmologists we have spoken to postconsensus felt that the volume needed to be kept low at 2 mL per injection attempt (and the concentration high). However, there is a counter view that high volumes are required to force hyaluronidase across components of the eye and 4 to 5 mL has also been suggested.^44,45^This was agreed to unanimously.

### Question 6: Should the Clinic Anticoagulate the Patient at the Bedside With Aspirin or Other Agents?

Should we as a consensus group be advocating anticoagulation at the bedside? It has been suggested in other consensus documents that anticoagulation should be given at the bedside.^23,46^ Suggested anticoagulation has included:

- Aspirin in varying doses of 125 to 650 mg stat
- Heparin or enoxaparin sodium
- It was felt by the consensus group and from further advice sought that in the case of hyaluronic acid-induced visual loss, the risks associated with anticoagulation were not outweighed by any potential benefit.

Retrobulbar or peribulbar injection of hyaluronidase may be riskier with an increased chance of retrobulbar hemorrhage.

It was agreed unanimously that there is little evidence for anticoagulating patients at the bedside. Because this is initially a nonthrombotic occlusion it is not advised.

#### Key Point

It is felt that anticoagulation should not be performed at the bedside.

### Question 7: Should the Clinic Attempt Maneuvers to Decrease Intraocular Pressure at the Bedside or During Transport?

Should we as a consensus group suggest maneuvers to decrease intraocular pressure? It has been suggested that the practitioner and patient may try to decrease intraocular pressure by the following means:

- Ocular massage
- Re-breathing in a paper bag or Carbogen (95%O^2^, 5% CO^2^)
- Timolol 0.5% drops (1-2 drops each eye)
- Sublingual glycerol trinitrate
- The majority of ophthalmologists asked postmeeting felt the above maneuvers, especially closed eye massaging, were worthwhile trying but unlikely to be successful (4/5). The minority view was for this not to be done at bedside.

#### Key Points

1. Attempts to decrease intraocular pressure were felt to have low levels of evidence
2. The consensus group did not reach agreement on the value of these maneuvers
3. However, the group did not recommend against attempts to decrease intraocular pressure at the bedside

However, there was no consensus reached on these series of maneuvers. The group considers there are not enough data on these points at this time to reach a conclusion as to their effectiveness.

## DISCUSSION

This consensus group is not the first to attempt to improve safety and introduce a template of management for this rare but devastating adverse event from tissue fillers.^19,25,35,47^ However, it was felt that this consensus group could add value to the literature by also defining a consent process and by suggesting preventative measures. This should begin with an understanding of the relative risk of each region in the face and extend into practical injection technique. There is a disparate approach in the literature to the obligations of the practitioner at the bedside, with different consensus documents and articles setting very different expectations. Because this complication may occur in a variety of settings and affect practitioners with a variety of skill levels, advice should be tailored to avoid issues of incorrect diagnosis and treatment by practitioners beyond their comfort level. It must be acknowledged that this issue is associated with extreme practitioner and patient anxiety, and therefore clear guidelines to follow would be helpful.

This group found unanimity of opinion in all but one of the issues raised for discussion. Specifically, terms of consent, techniques for safest injection technique, division of facial regions along lines of safety for teaching purposes, documentation and examination at the bedside of the event, hyaluronidase utilization at injection site (in the case of hyaluronic acid embolization), and anticoagulation (not to use) were agreed on. Only in the use of agents to decrease intraocular pressure was there an absence of consensus largely because the group felt there was a lack of data to support a position either way.

Limitations to this consensus document include the incomplete data and evidence-based reports that were accessible to the authors. Issues included the rarity of the event and the inability to access any prospective studies in vivo in humans given the nature of the condition. Limitations in studies also impacted the possible validity of some of our safety recommendations such as cannulae being safer in certain scenarios, utilizing local anesthetic before cannula use, the direction of the instrument, and injecting away from the eye. We had insufficient data on the utility of procedures to lower intraocular pressure in the event of embolic events. We also felt in the light of incomplete evidence surrounding the successful utilization of retrobulbar injection of hyaluronidase that we could not recommend this as treatment at the bedside except in those experienced with its utilization.

## CONCLUSIONS

The major conclusions of this consensus are:

1. The patient should be adequately consented for the possibility of visual impairment and cerebrovascular accident, and the patient should also agree to remedial measures in the event of these adverse events.
2. Safety measures should be employed in all patients including understanding the injection anatomy in any given area, injecting slowly with low extrusion pressure, understanding that cannulae are safer in some areas but not all areas, considering local anesthetic with epinephrine at cannula entrance points, directing the injection instrument across axial vessels, utilizing micro boluses (<0.1 mL), continually moving the instrument even in small amplitude movements in the chosen plane, and injecting in a direction away from the eye when possible.
3. There is currently no evidence to support aspiration as a safety measure, and the consensus was against relying on this maneuver.
4. It is possible to grade regions according to risk and the clinical environment in which these injections should be delivered. The forehead, nose, and glabella were considered the regions that should only be treated by those with extensive experience, comprehensive training, and up-to-date training in anatomy and safest practice and delivered in a medical clinic where help may be readily available.
5. If visual loss is suspected, this should be documented as should cutaneous and neurological involvement.
6. All clinics should be equipped with hyaluronidase and know how to utilize this for cutaneous involvement and attempt injection at the suspected injection point of filler as well as supratrochlear and/or supraorbital regions.
7. Retrobulbar and peribulbar hyaluronidase should be attempted only by those experienced in this technique.
8. Anticoagulation should not be performed at the bedside.
9. The consensus group could not agree on the effectiveness of maneuvers to decrease intraocular pressure and took no stance on these.
